# Advances in research on the anti-tumor mechanism of *Astragalus* polysaccharides

**DOI:** 10.3389/fonc.2024.1334915

**Published:** 2024-03-06

**Authors:** Qian Yang, Dandan Meng, Qinyuan Zhang, Jin Wang

**Affiliations:** School of Chinese Medicine, Shandong University of Traditional Chinese Medicine, Jinan, China

**Keywords:** Astragalus polysaccharides, anti-tumor therapy, cancer, anti-tumor mechanism, apoptosis, autophagy

## Abstract

The dry root of the soybean plant *Astragalus membranaceus* (Fisch) Bge. var. *mongholicus* (Bge) Hsiao or *A. membranaceus* (Fisch) Bge, Astragali Radix (AR) has a long medicinal history. *Astragalus* polysaccharide (APS), the natural macromolecule that exhibits immune regulatory, anti-inflammatory, anti-tumor, and other pharmacological activities, is an important active ingredient extracted from AR. Recently, APS has been increasingly used in cancer therapy owing to its anti-tumor ability as it prevents the progression of prostate, liver, cervical, ovarian, and non-small-cell lung cancer by suppressing tumor cell growth and invasion and enhancing apoptosis. In addition, APS enhances the sensitivity of tumors to antineoplastic agents and improves the body’s immunity. This macromolecule has prospects for broad application in tumor therapy through various pathways. In this article, we present the latest progress in the research on the anti-tumor effects of APS and its underlying mechanisms, aiming to provide novel theoretical support and reference for its use in cancer therapy.

## 1 Introduction

According to the 2020 Global Cancer Statistical Report, cancer is the second leading cause of death globally, with an increase in incidence in recent years (1, 2). Malignant tumors are the primary reason for chronic noninfectious disease-related deaths and are the chief obstacle to a better life expectancy (3). Both gene mutation and epigenetic changes are important factors that lead to cancer occurrence and progression (4). Epigenetics refers to the reversible and heritable changes in gene function without changes in the nuclear DNA. Epigenetic changes include histone modification, DNA methylation, chromatin remodeling, and non-coding RNA regulation, which can affect the expression and silencing of oncogenes and tumor suppressor genes, respectively (4–7). Therefore, epigenetic regulation offers a promising direction and strategy for the development of anti-tumor therapeutics. However, as the pathogenesis of cancer remains unclear, the development of effective treatment methods is limited. Currently, cancer treatment primarily involves surgical resection, radiotherapy, chemotherapy, or a combination of two or all three approaches. During treatment, both cancerous and normal cells are killed, triggering a series of adverse reactions and toxic side effects with varying degrees of impact on prognosis and patients’ quality of life (QOL). Recently, with advancements in tumor microenvironment research, immunotherapy has emerged as a promising therapeutic approach (8, 9). Tumor immunotherapies, such as the use of immune checkpoint inhibitors (targeting programmed cell death protein-1, programmed cell death ligand-1, cytotoxic T lymphocyte-associated antigen 4, etc.) and adoptive cell transfer (such as chimeric antigen receptor T-cell therapy, T cell receptor-engineered T cells therapy, tumor-infiltrating lymphocytes therapy, etc.) (10–13), have demonstrated promising outcomes; however, their efficacy must be validated using clinical trial.

Traditional Chinese medicine has been essential in tumor prevention and management, with significant advantages, particularly in improving and managing clinical symptoms, enhancing the efficacy of anti-cancer drugs, reducing the toxicity of radio- and chemotherapies, enhancing the patient’s immunity and QOL, and effectively prolonging survival time (14), making it a critical strategy for tumor prevention and management. Astragali Radix (AR), a common traditional Chinese herbal medicine used in China for over 2,000 years, is also extensively used in numerous countries and has been included in the pharmacopeias of the United States, Japan, and South Korea (15). Medical research shows that AR exhibits various therapeutic activities, including anti-tumor (16–19), anti-aging (20), immune regulatory (21), anti-fibrosis (22), antibacterial (23), antiviral (24, 25), and anti-radiation (26) effects. *Astragalus* polysaccharide (APS), a major active component of AR (27–30), is extensively used in medicine as it demonstrates beneficial activities and low toxicity (31).

APS is a mixture of macromolecules, mainly composed of glucans (both water-soluble and water-insoluble) and heteropolysaccharides (mostly water-soluble and acidic), with molecular weights ranging from 8.7×10^3^ to 4.8×10^6^ Da (31–34). According to recent pharmacological studies, APS has a wide spectrum of biological activities, such as anti-inflammatory (35–37), immune regulation (38, 39), anti-fiber (40), anti-radiation (41, 42), anti-aging (21), anti-metabolic disorders (43), protective effects on the cardiovascular system (44, 45), anti-diabetes (46), anti-tumor (47, 48), and anti-infection (49).

Owing to its high efficacy and low toxicity, the role and value of APS in the management of malignancies have garnered increasing research attention in recent years. The anti-cancer mechanism of APS involves inducing the apoptosis of tumor cells by regulating various pathways; inhibiting proliferation, migration, and invasion of tumor cells; regulating immune function and autophagy; and enhancing the efficacy of chemotherapeutic or targeted drugs by reducing their toxicity. However, a comprehensive understanding of how APS works has not been put into perspective. In this study, the anti-tumor mechanisms of APS and its targets are comprehensively reviewed to render a new theoretical basis for cancer treatment.

## 2 Anti-tumor mechanism of APS

### 2.1 Role of APS in inducing tumor cells apoptosis

Apoptosis, also referred to as programmed cell death, plays a key role in maintaining internal environment homeostasis (50, 51). Induction of cell death is a major mechanism underlying the activity of anti-tumor drugs (52).

#### 2.1.1 B-cell lymphoma-2 (Bcl-2) family

The Bcl-2 family contains a class of molecules involved in apoptosis-associated pathway modulation. Bcl-2 is an anti-apoptosis gene highly expressed in various tumors (53, 54), whereas Bcl-2-associated X (Bax) is a pro-apoptotic gene (55). Huang et al. proposed that APS induces H22 (a hepatocellular cancer [HCC] cell line) apoptosis by downregulating Bcl-2 and upregulating Bax expression (56). Similarly, Lv et al. reported that after APS treatment, the apoptosis of HepG2 cells is accelerated (57). Specifically, APS decreased the levels of Bcl-2, β-catenin, c-myc, and Cyclin D1 in cells, suggesting that the mechanism of tumor suppression may be related to the inhibition of Bcl-2 expression by downregulating the Wnt/β-catenin signaling pathway. Xie et al. provided additional data demonstrating that APS effectively inhibited the growth of MDA-MB-231 (a human breast cancer [BC] cell line) graft tumor (58). In terms of mechanism, APS concentration-dependently increased Bax protein expression and decreased Bcl-2 protein expression, thus inducing MDA-MB-231 apoptosis.

#### 2.1.2 miRNA pathway

miRNAs, endogenous non-coding small RNAs that are critical in regulating almost all signaling pathways in eukaryotic cells (59), have recently been found to participate in tumor occurrence and progression by regulating apoptotic signaling pathways (60–63). miR-27a is over-expressed in various tumor cells (64–66). Guo et al. found that APS significantly and dose-dependently reduced miR-27a levels in cells, subsequently upregulating the expression of the tumor suppressor gene *FBXW7*, thereby inhibiting OV-90 and SKOV-3 proliferation and significantly increasing apoptosis (67). miR-133a, which inhibits cancer cell growth in multiple tumors, is considered a tumor suppressor molecule (68–70). According to Chu et al., after APS treatment, the apoptosis rate of human osteosarcoma MG63 cells increased owing to the upregulation of miR-133a and inactivation of the JNK signaling pathways (71).

#### 2.1.3 Extrinsic apoptosis pathways

Extrinsic apoptosis, one of the major pathways of apoptosis, is mediated by death receptors on the cell surface (72). The binding of Fas ligands to Fas receptors or tumor necrosis factor (TNF) receptors to TNF ligands is the primary way of initiating this apoptotic pathway (73–75). Fas and its ligands, a class of important apoptosis-inducing molecules, exist in various tumor cells (76, 77). In an *in vitro* study of colon cancer CD133^+^/CD44^+^ cells, Li and Shen found that APS can induce apoptosis by activating the Fas death receptor pathway. Specifically, APS increases Fas expression and induces apoptosis in a concentration-dependent manner (78).

#### 2.1.4 p53 protein

The p53 protein is a vital tumor suppressor, and its loss of function is a prerequisite for cancer development (79). In various cancers, p53 is a dominant force promoting apoptosis, cell cycle arrest, and DNA repair (80–83). Zhang et al. showed that APS could activate p53 and p21 and inhibit the expression of Notch1 and Notch3 *in vitro*, ultimately inhibiting cell proliferation and promoting their apoptosis (84) (**Table 1**).

**Table 1:** Effects of APS on the signaling pathways of apoptosis.

Test type	Cancer types	Cell type/Animal model	Dosage/concentrations	Signaling pathways (↑upregulation, ↓downregulation)	REF
***in vitro***	Hepatocellular carcinoma	H22 cells	0.1, 0.5, 1 mg/mL	Bcl-2↓ and Bax↑	(56)
	Liver cancer	HepG2 cells	100, 200 mg/L	Bcl-2↓, β-catenin↓, c-myc↓, Cyclin D1↓ and Wnt/β-catenin↓	(57)
	Ovarian cancer	OV-90 cells and SKOV-3 cells	0–2 mg/mL	miR-27a↓ and FBXW7↑	(67)
	Osteosarcoma	MG63 cells	10 mg/mL	miR-133a ↑and JNK↓	(71)
	Colon cancer	RKO cells	12.5, 25, 50 mg/mL	Fas↑	(78)
	Non-small cell lung cancer	H460 cells	0–30 mg/mL	p53↑, p21↑, Notch1↓and Notch3↓	(84)
***in vivo***	Breast cancer	BALB/c-nu nude mice♀, MDA-MB-231 cells	200, 400 mg/kg bw	Bax↑ and Bcl-2↓	(58)

Bcl-2, B-cell lymphoma-2; Bax, Bcl-2-associated X; Cyclin D1, G1/S-Specific cyclin-D1; c-myc, Myelocytomatosis viral oncogene homolog; Wnt/β-catenin , Wnt/β-catenin signaling pathway; FBXW7, F-box/WD repeat-containing protein 7; JNK, c-Jun N-terminal kinases; Fas, Fas transmembrane glycoprotein; p53, p53 protein; p21, p21 protein.

### 2.2 Suppression of cancer cell proliferation

Tumors promote abnormal cell proliferation and metabolic activity by disrupting the regulation of growth-promoting signals (85); therefore, suppressing tumor cell proliferation is a vital strategy in the treatment of tumors.

#### 2.2.1 Promotion of cell cycle arrest

Cell cycle regulation is coordinated by a complex network of interactions between enzymes, cytokines, and cell cycle signaling pathways. This regulation is essential for cell proliferation, growth, and repair (86). Abnormal regulation of the cell proliferation cycle is a major cause of tumor initiation (87). In an *in vitro* experiment, by inhibiting the JAK2/STAT3 pathway, Liu et al. found that APS induced the cell cycle of bladder cancer UM-UC-3 to stop in the G0/G1 phase, thus inhibiting its proliferation (88). Additionally, Yu et al. provided new evidence demonstrating that APS promotes mouse solid tumor S180 cell apoptosis in a dose-dependent manner through S-phase arrest (89). An *in vitro* study of APS against the proliferation of HCC cells established that APS increased the G1 phase arrest of human hepatoma HepG2 cells, enhanced their autophagic activity, suppressed proliferation, and enhanced apoptosis by inhibiting the AKT axis (90). Yan et al. showed that APS could inhibit the proliferation of colon cancer SW620 cells through cell cycle arrest, with G2/M phase arrest playing a dominant role (91).

#### 2.2.2 Others

Abnormal activation of cell signal transduction pathways is closely related to the occurrence and development of malignant tumors (92, 93). In an *in vitro* experiment, APS was found to inhibit RT4 and T24 proliferation and migration by inducing the accumulation of Fe^2+^ and malondialdehyde in cells, and ferrostatin, an iron ptosis inhibitor, reversed this reaction (94). Furthermore, APS was found to reduce GPX4 expression, inhibit the activity of the light chain subunit SLC7A11 (xCT), and promote the formation of BECN1-xCT complex by activating AMPK/BECN1 signaling. These findings demonstrated the ability of APS to inhibit urothelial carcinoma progression by inducing iron ptosis. According to Guo et al., APS attenuated the proliferative and invading capacities of prostate cancer cells (PC3 and DU145) *in vitro* and inhibited PC3 xenograft growth *in vivo*, time- and dose-dependently (95). Moreover, APS significantly inhibited tumor development by upregulating miR-138-5p expression and inhibiting SIRT1 and SREBP1 expression. Furthermore, APS significantly suppresses HeLa cell growth, invasion, and migration (96). This may be achieved by increasing SHP2 and SOCS3 protein levels in cells and inhibiting JAK-STAT pathway overactivation. Wu et al. demonstrated that APS could control the proliferation of lung cancer cells (A549 and NCI-H358 cells) by inhibiting the NF-κB signaling pathway (97) (**Table 2**).

**Table 2:** Effects of APS on the signaling pathways of proliferation.

Test type	Cancer types	Cell type/Animal model	Dosage/concentrations	Signaling pathways (↑upregulation, ↓downregulation)	REF
***in vitro***	Bladder cancer	UM-UC-3 cells	500, 1000 mg/mL	G0/G1 phase arrest and JAK2/ STAT3↓	(88)
	Liver cancer	HepG2 cells	25, 50, 100 μg/mL	G1 phase arrest, AKT↓ and p-AKT↓	(90)
	Colon cancer	SW620 cells	1 g/L	G2 /M phase arrest	(91)
	Urothelial carcinoma	RT4 cells and T24 cells	10, 15 μm	Fe^2+^↑, BECN1-xCT↑, GPX4↓, xCT↓ and AMPK/BECN1↑	(94)
	Prostate cancer	PC3 cells and DU145 cells	0-40 mg/mL	miR-138-5p↑, SIRT1↓ and SREBP1↓	(95)
	Cervical cancer	Hela cells	0–16 mg/mL	SHP2↑, SOCS3↑ and JAK-STAT↓	(96)
	Non-small cell lung cancer	A549 cells and NCI-H358 cells	20, 40 mg/mL	NF-κB↓	(97)
***in vivo***	Ascites tumor	Kunming mice , S180 cells	150, 300 mg/kg bw	S phase arrest	(89)

JAK2, anus kinase 2; STAT3, Signal transducer of activators of transcription; AKT, Protein kinase B; p-AKT, phospho- Protein kinase B; BECN1, Beclin-1; GPX4, Glutathione peroxidase 4; xCT, Light chain subunit SLC7A11; AMPK, Adenosine monophosphate activated protein kinase; SIRT1, Silent mating type information regulation 2 homolog- 1; SREBP1, Recombinant Sterol Regulatory Element Binding Transcription Factor 1; SHP2, SH2 domain-containing protein tyrosine phosphatase 2; SOCS3, Suppressor of cy-tokine signaling proteins; NF-κB, Nuclear factor kappa-B.

### 2.3 Role of APS in inhibiting tumor invasion and metastases

Tumor invasion and metastases are strongly correlated with adverse prognoses, and the invasion of tumor cells is a prerequisite for metastases. Tumor metastasis is a complicated process involving multiple stages, genes, and gene products. Inhibiting tumor metastasis can prevent tumor cell spread to other body sites, thereby mitigating tumor progression (98).

#### 2.3.1 Epithelial–mesenchymal transition (EMT) pathway

Multiple studies have linked EMT to tumor progression, invasion, and metastases (99, 100). Yang et al. found through *in vitro* experiments that APS significantly prevents human BC cells, MCF-7 and Mda-MB-231, from invasion and migration (101). Further research demonstrated the ability of APS to inhibit BC cell invasiveness and migration by regulating the Wnt/β-catenin axis. In addition, macrophage migration inhibitory factor (MIF), a pro-inflammatory factor that is critical in the onset and progression of intestinal, breast, and prostate carcinoma among other malignant tumors (102–104), induces EMT in cancer cells (105, 106). Liao et al. found that an injectable preparation of APS (PG2) dose-dependently inhibited the migratory and invasive activities of lung adenoma A549 cells (107). For specific performance, APS treatment led to reduced EMT markers (vimentin, AXL) and MIF levels in cells.

#### 2.3.2 Regulation of miRNA expression

miRNAs contribute to the proliferation, invasion, and migration of tumor cells by regulating gene transcription (108) and play a key regulatory role in the pathological process of various human tumors. *In vitro* experiments utilizing A549 and NCI-H1299 by Tao et al. substantiated that APS treatment markedly attenuated the migration and invasiveness of non-small-cell lung cancer (NSCLC) cells compared to that seen with the control, and the underlying mechanism may be related to the APS-related increase in cellular miR-195-5p levels (109). Notably, the increased expression of miR-133a in cells after APS treatment effectively prevents the proliferation, migration, and invasion of prostate cancer cells (DU145) (110).

#### 2.3.3 Vascular endothelial growth factor (VEGF) pathway

Tumor angiogenesis is the direct path of tumor cell metastasis (111). VEGF can promote tumor progression in patients with cancer by regulating angiogenesis in cancer tissues (112, 113). Recent studies have shown that the downregulation of VEGF is a positive signal during tumor therapy (114). Zhao et al. reported that APS inhibits Lewis lung cancer growth and metastasis in mice by significantly reducing VEGF and EGFR expression in cancerous tissues (115). Additionally, *in vitro* studies by Tang and Li demonstrated that APS inhibits the metastasis of gastric cancer cells (SGC7901) induced by vascular endothelial cells (HUVECs) (116) (**Table 3**).

**Table 3:** Effects of APS on the signaling pathways of invasion.

Test type	Cancer types	Cell type/Animal model	Dosage/concentrations	Signaling pathways (↑upregulation, ↓downregulation)	REF
***in vitro***	Breast cancer	MCF-7 cells and Mda-MB-231 cells	200, 400, 800 μg/mL	Cyclin D1↓, c-myc ↓and Wnt/β-catenin↓	(101)
	Non-small cell lung cancer	A549 cells and NCI-H1299 cells	5, 10, 20 μg/mL	miR-195-5p↑	(109)
	Prostate cancer	DU145 cells	1, 2.5, 5 mg/mL	miR-133a↑	(110)
	Gastric cancer	SGC7901 cells	2.5, 5, 10, 20, 40 mg/mL	E-cadherin↑, Vimentin↓, MMP-13↓ and MMP-9↓	(116)
***in vivo***	Lung cancer	C57BL/6J mice and lung cancer Lewis tumor cells	25, 50, 100 mg/kg bw	VEGF↓ and EGFR↓	(115)
***in vitro* and *in vivo***	Adenocarcinoma of lung	A549 cells; NOD/SCID mice♂	0-1000μg/mL; 10, 40, 160 mg/kg bw	E-cadherin↑, Vimentin↓, AXL↓ and MIF↓	(107)

Cyclin D1, G1/S-Specific cyclin-D1; c-myc, Myelocytomatosis viral oncogene homolog; Wnt/β-catenin , Wnt/β-catenin signaling pathway; MMP-13, Matrix metalloproteinase-13; MMP-9, Matrix metalloproteinase-9; VEGF, Vascular endothelial growth factor; EGFR, Epidermal growth factor receptor; MIF, Macrophage migration inhibitor factor; AXL, Recombinant AXL Receptor Tyrosine Kinase.

### 2.4 Nano-drug delivery systems can increase efficiency and reduce toxicity

APS is a mixture of hydrophilic macromolecules (117) that cannot easily penetrate cell membranes, with only a small portion absorbed through the intercellular space (118), limiting its clinical application. Nano-drug delivery systems are highly selective and can deliver drugs to specific sites to enhance therapeutic effects and reduce adverse reactions (119, 120). Therefore, they show great potential for the development and application of anti-tumor drug delivery to improve the therapeutic effects.

Selenium nanoparticles, by virtue of having high bioavailability, potent bioactivity, and low toxicity (121–123), exert significant inhibitory effects on various malignant tumors (124–126). Ji et al. prepared a novel functionalized nanocomposite using alcohol-soluble APS and selenium nanoparticles and found that it was effective in suppressing HepG2 proliferation and accelerating apoptosis by triggering S-phase arrest, thereby stimulating ΔΨm (mitochondrial membrane potential) depletion, increasing the Bax/Bcl-2 ratio, and promoting intracellular reactive oxygen species accumulation (127). Jiao et al. developed selenium nanoparticles modified with macromolecular weight APS and observed positive results in hepatoma treatment, as indicated by the induction of apoptosis and inhibition of proliferation of HepG2 cells (128). The mechanism involved may be related to the increasing S-phase block, the significant enhancement of Bax levels, and the marked reduction of Bcl-2 levels and ΔΨm value. Studies have shown that the selenium nanoparticles modified by APS are cytotoxic to MCF-7 cells. This cytotoxicity is achieved by the induction of apoptosis through the mitochondrial pathway and the activation of autophagy at an early stage and inhibiting it at a late stage (129). Moreover, Huang et al. successfully constructed APS superparamagnetic iron-oxide nanocomposites and demonstrated that they could effectively induce M1 polarization of mouse monocytic macrophage RAW264.7 and improve the killing ability of macrophages against HepG2 cells *in vitro*. Furthermore, no inhibitory effect on macrophage proliferation was observed (130).

### 2.5 Combination of APS with anti-tumor drugs improves effectiveness and reduces toxicities

Resistance to chemotherapy is the primary reason for treatment failure and poor prognoses. Therefore, focusing on the mechanism of drug resistance and inhibiting it in tumor cells is essential to reducing drug resistance and optimizing effectiveness while improving patient survival rates.

#### 2.5.1 Cisplatin (CDDP)

CDDP is widely used as an initial medication in cancer therapy owing to its exceptional ability to combat cancer and its broad spectrum of effectiveness against various cancer types. However, the prolonged utilization of this drug may result in the development of resistance, thereby restricting its practical implementation in clinical settings. The PI3K/AKT axis is crucial for tumorigenesis and progression (131). As indicated by Gong et al., APS reversed the acquired CDDP resistance in melanoma cell lines B16 *in vivo* by inhibiting the PD-L1/PI3K/AKT axis (132). Liu and Chen showed that APS was able to overcome the resistance of A549/CDDP cells to CDDP *in vitro* (133). Subsequent investigations revealed that APS can decrease the ΔΨm values and Bcl-2, p-PI3K, P-gp, and p-AKT levels while elevating Bax expression. This finding implies that the potential mechanism of action could encompass the inhibition of the PI3K/AKT pathway and stimulation of the mitochondrial apoptosis pathway. Lu et al. further demonstrated that APS combined with CDDP effectively inhibits proliferation, migration, and EMT progression of CDDP-resistant SW620 cells by inhibiting miR-10b-5p expression and upregulating AGPAT3 expression (134). The combination of APS and CDDP synergistically inhibits the invasion and metastasis of CNE-1 (a human nasopharyngeal carcinoma cell line). It is associated with the induction of G0/G1 and S phase arrest, downregulation of MMP-9 expression, and upregulation of p53 expression (135). Li et al. observed that APS could enhance the sensitivity of SKOV3 ovarian cancer cells to CDDP treatment by activating the mitochondrial apoptosis pathway and JNK1/2 signaling pathway (136).

#### 2.5.2 Other drugs

In addition to CDDP, the application of APS can potentially augment the chemosensitivity of tumors to other medications. Li et al. showed that APS enhanced the sensitivity of HCC cells to doxorubicin chemotherapy and induced cancer cell apoptosis (137). *In vitro* tests have shown that by downregulating OGT (O-GlcNAc transferase) and upregulating OGA (O-GlcNAc transferase) expression in Hep3B cells, APS reduced O-GlcNAcylation and intensified endoplasmic reticulum stress responses. According to *in vivo* experiments, APS combined with doxorubicin inhibited xenograft tumor growth in mice, suggesting that APS can potentially be an optional sensitizer in HCC chemotherapy. A previous study demonstrated that APS effectively reversed the resistance of lung adenocarcinoma (PC9 and HCC827) cells to gefitinib by inhibiting the PD-L1/SREBP-1/EMT axis (138). In a mouse BC model, Bao et al. (139) found that APS effectively alleviated paclitaxel-induced cytotoxicity in mouse monocyte–macrophage RAW264.7 cells. The mechanism for this action is attributable to changes in the cell cycle and apoptosis. When studying the role of APS plus apatinib in human pancreatic cancer cell (ASPC-1 and PANC-1) proliferation and apoptosis, Wu et al. found that the combination therapy contributed to higher cell migration and invasion inhibition rates and apoptosis and lowered p-AKT, MMP-9, and p-ERK levels than those in the control group (140), suggesting that APS plus apatinib is a promising strategy for treating pancreatic cancer (**Table 4**).

**Table 4:** APS acts synergically with other chemotherapeutic drugs.

Test type	Standard anti-cancer drugs	Cancer types	Cell type/Animal model	Dosage/ concentrations	Signaling pathways (↑upregulation, ↓downregulation)	REF
***in vitro***	Cisplatin	Lung cancer	A549/CDDP cells	100 mg/L	Bax↑, ΔΨm↓, Bcl-2↓, P-gp↓, and PI3K/AKT↓	(133)
		Colorectal cancer	SW620 cells, SW620/CDDP cells	1 mg/mL	miR-10b-5p↓ and AGPAT3↑	(134)
		Nasopharyngeal carcinoma	CNE-1 cells	200 μg/mL	MMP-9↓, p53↑, G0/G1 phase arrest and S phase arrest	(135)
		Ovarian cancer	SKOV3 cells	800 μg/mL	Bcl-2↓, Bax↑, caspase-3↑, and JNK1/2↑	(136)
	Gefitinib	Adenocarcinoma of lung	PC9 cells and HCC827 cells	200 mg/L	PD-L1/SREBP-1/EMT↓	(138)
	Apatinib	Pancreatic cancer	ASPC-1 cells and PANC-1 cells	200 μg/mL	p-AKT↓, p-ERK↓ and MMP-9↓	(140)
***in vivo***	Taxol	Breast cancer	4T1 cells, mouse mononuclear macrophage RAW264.7 and BALB/C mice	40 mg/kg bw	G2/M phase arrest↓, Taxol-induced cytotoxicity↓	(139)
***in vitro* and *in vivo***	Cisplatin	Melanoma	A375/CDDP cells, B16/CDDP cells; C57BL/6/SCID mice♂	200 mg/kg bw	PD-L1/PI3K/AKT↓	(132)
	Doxorubicin	Hepatocellular carcinoma	Hep3B cells; BALB/c nude mice♂	0-50 mg/L	OGT↓, OGA↑, O-GlcNAcylation↓	(137)

Bax, Bcl-2-associated X; ΔΨm, Mitochondrial membrane potential; Bcl-2, B-cell lymphoma-2; PI3K, Phosphatidylinositol-3-kinase; P-gp, P-glycoprotein; AKT, Protein kinase B; Fas, Fas transmembrane glycoprotein; AGPAT3, 1-acylglycerol-3-phosphate O-acyltransferase 3; MMP-9, Matrix metalloproteinase-9; p53, p53 protein; JNK, c-Jun N-terminal kinases1/2; PD-L1, Programmed cell death protein-ligand 1; SREBP1, Recombinant Sterol Regulatory Element Binding Transcription Factor 1; EMT, Epithelial-mesenchymal transition; p-AKT, phospho-Protein kinase B; p-ERK; phospho-Extracellular Regulated Protein Kinases; OGT, O-GlcNAc transferase; OGA, O-GlcNAcase;O-GlcNAcylation, Methods to detect the expression of O-GlcNAc.

### 2.6 Immunomodulation

The body’s immune system, through various pathways, identifies and removes mutated tumor cells under normal circumstances, which inhibits tumor cell growth to some extent. Recent evidence has indicated that immune suppression in the microenvironment before tumor metastasis is a key link in the initiation of tumor metastasis (141). Owing to its strong immunomodulating properties (142), APS can enhance the body’s immunity to treat various cancers.

Bamodu et al. reported that APS can downregulate the expression of interleukin (IL)-6/10, markedly increase the M1/M2 macrophage polarization ratio, contribute to the functional maturity of DC, enhance T cell-medicated anti-tumoral immune responses, improve the accuracy of tumor cell killing, and inhibit the growth of tumor cells (143). Wei et al. reported that, through the Notch signaling pathway, APS significantly promoted the production of cytokines such as IL-6 and TNF-α; increased the iNOS levels and polarization rate of M1/M2 macrophages; activated M1 macrophages; and inhibited M2 macrophages, thereby enhancing the killing and phagocytosis of tumor 4T1 cells and the inhibition of tumor growth and metastasis (144). Li et al. observed that the presence of APS can elevate the percentage of M1 macrophages within liver cancer tissues while simultaneously reducing the proportion of M2 macrophages, thereby inhibiting the growth of liver cancer tumor cells (145). Furthermore, APS activates the release of NO and TNF-α by macrophages, thus reinforcing the suppressive and killing impact of the immune system on MCF-7 BC cells (146).

Ding et al. showed that APS inhibits tumor growth in melanoma-bearing mice (147). Specifically, by regulating the composition of the intestinal flora and altering fecal metabolites, APS reduces the MDSC (Myeloid-derived suppressor cell) count, downregulates IL-10, arginase-1, and TGF-β expression, and decreases the immunosuppressive activity of MDSCs in mice with melanoma, thereby enhancing the killing ability of CD8^+^ T cells on tumors. Yu et al. prepared a novel APS using water at 4°C (148) and demonstrated that it activated anti-tumoral immune responses and enhanced anaerobic metabolism in the solid tumor microenvironment through the HIF-1 axis, ultimately promoting mouse S180 (a cancer cell line) apoptosis (89). He et al. injected APS into HCC BALB/c mice (100, 200, and 400 mg/kg per day for 12 consecutive days) and observed increased CD8^+^ T cell count, decreased PD-L1 levels, and reduced tumor size, weight, and volume. Furthermore, by upregulating miR-133a-3p and downregulating MSN, APS attenuated PD-L1-mediated immunosuppression, thereby suppressing tumors (149). Chang et al. found that APS downregulates PD-L1 protein levels by inhibiting the AKT/mToR/p70S6K axis, thereby enhancing the immune capacity of 4T1 (mouse BC) and CT26 (mouse colorectal cancer) cells (150) (**Table 5**).

**Table 5:** Immunomodulatory effects of APS.

Test type	Cancer types	Cell type/Animal model	Dosage/concentrations	Signaling pathways (↑upregulation, ↓downregulation)	REF
***in vitro***	Breast cancer	MCF-7 cells and RAW264.7 cells	200-1000 μg/mL	TNF-α↑and NO↑	(146)
***in vivo***	Melanoma	B16-F10 cells and C57BL/6 mice♂	200 mg/kg bw	MDSC↓, Arginase-1↓, interleukin-10↓, transforming growth factor-β↓ and CD8^+^T cells↑	(147)
	Ascites tumor	Kunming mice, S180 cells	150, 300 mg/kg bw	HIF-1↑, CD3^+^ cells↑, CD4^+^ cells↑ and CD8^+^T cells↑	(89)
***in vitro* and *in vivo***	Non-small cell lung cancer	H441 cells, H299 cells, LLC1 cells; C57BL/6 mice♂	16 mg/ml; 3 mg/kg bw	IL-6/10↓, M1/M2 macrophage polarization ratio↑ and DC functions mature↑	(143)
	Breast cancer	4T1 cells, RAW264.7 cells; BALB/c mice♀	30, 100, 300 μg/mL	IL-6↑, TNF-α↑, iNOS↑ and M1/M2 macrophage polarization ratio↑	(144)
	Hepatocellular carcinoma	MHCC97H cells, Huh7 cells, THP1 cells; BALB/c nude mice♂	8, 16 mg/mL; 50, 100, 200 mg/kg bw	M1 macrophages↑ and M2 macrophages↓	(146)
	Hepatocellular carcinoma	SMMC-7721 cells, Huh7 cells; BALB/c mice	0.1, 0.5, 1 mg/mL; 100, 200, 400 mg/kg bw	CD8^+^T cells↑, PD-L1↓, miR-133a-3p↑ and MSN↓	(149)
	Breast cancer; Colon cancer	4T1 cells and CT26 cells; BALB/c mice	10,000 ng/ml; 50 mg/kg bw	PD-L1↓ and AKT/mTOR/p70S6K↓	(150)

TNF-α, Tumor necrosis factor-α; NO, Nitric oxide; MDSC, Myeloid-derived suppressor cells; HIF-1, Hypoxia-inducible factor-1; IL-6/10, Interleukin-6/10; IL-6, Interleukin-6; iNOS, Inducible nitric oxide synthase; PD-L1, Programmed cell death protein-ligand 1; MSN, Moesin; AKT, Protein kinase B; mTOR, Mammalian rapamycin target protein; P70S6K, P70 ribosomal protein S6 kinase.

Chang et al. revealed that APS enhances immune responses in 4T1 and CT26 tumor-bearing mice by downregulating PD-L1 protein levels by inhibiting the AKT/mTOR/p70S6K axis (140).

### 2.7 Other anti-tumorigenic effects of APS

#### 2.7.1 Lipid metabolism

Lipid metabolism is a major pathway of cellular energy metabolism. Abnormal lipid metabolism can promote tumor progression (151, 152), which is an indicator of human cancer (153).

Lipid metabolism is a complex regulatory process that provides energy, lipid chains, and a large amount of fats required for the formation of new cell membranes for rapidly dividing and proliferating cancer cells (154). Abnormal lipid metabolism is often accompanied by the anomalous overexpression of related enzymes (155), abnormal transcription of related non-coding RNA (156), and activation of carcinogenic signaling pathways (157). Cholesterol and its metabolites are signaling molecules that promote tumor development (158). Triglycerides are closely related to the growth of various tumor cells (159, 160). APS effectively lowers cholesterol and triglycerides (161, 162).

As indicated by a recent study, APS inhibits prostate cancer growth and lipid metabolism *in vivo* and *in vitro* (95). By upregulating miR-138-5p, APS significantly suppressed SIRT1 and SREBP1 expression, decreased cholesterol and triglyceride levels in PC3 and DU145, and attenuated cell proliferation. Therefore, the role played by APS in mediating lipid metabolism is important for the prevention of cancer progression.

#### 2.7.2 Autophagy

Autophagy refers to the biological process by which cells undergo intracellular degradation via lysosomes to protect cell integrity and maintain homeostasis under the influence of external environmental stimuli and metabolic pressures (163, 164). It is strongly linked to carcinogenesis and progression of various cancers and plays a dual role in the tumor process (165–167).

Zhi et al. found that after APS treatment, the expression of LC3B-II/I was significantly increased in colorectal cancer HCT-116 cells, while the expression levels of p-PI3K/PI3K, p-AKT/AKT, p-mTOR/mTOR, and p62 were significantly decreased (168). Therefore, they proposed that APS can induce autophagy in colorectal cancer cells by inhibiting the PI3K/AKT/mTOR axis and the development of cancer cells. The proportions of Beclin1 and LC3B in EC109 esophageal cancer cells increased significantly after APS treatment (169). Based on these findings, Chang et al. believed that the anti-tumor mechanism of APS was related to the enhancement of autophagy induced by APS. Li and Shen found that APS elevated caspase-9, caspase-3, and Bax protein levels, decreased Bcl-2 protein expression, and inhibited CD133 and CD44 co-positive colon cancer stem cell proliferation time- and concentration-dependently (78). Moreover, APS concentration-dependently induced apoptosis and this effect was reversed by an autophagy antagonist. These results suggest that APS can inhibit proliferation and promote apoptosis by inducing autophagy in colon cancer stem cells. However, given the dual role of autophagy in tumorigenesis and cancer progression, its exact mechanism in cancers requires further exploration.

### 2.8 Clinical trials on the anti-tumor effect of APS

The elucidation of the efficacy, safety, and tolerability of drugs administered to patients is the main focus of a clinical trial.

PG2 has recently been found to normalize the neutrophils-to-lymphocytes ratio in patients with lung cancer on combined immune checkpoint inhibitor therapy, suggesting that APS could be used to supplement anti-tumor agents (170). A trial of PG2 plus cytokine-induced killer cells for advanced NSCLC demonstrated that PG2 notably increased the proportion of peripheral blood CD4^+^ and CD3^+^ T lymphocytes (171), thus significantly improving patients’ functional status and relieving symptoms of qi deficiency, with remarkable clinical safety. A clinical study investigating PG2 plus gefitinib therapy for advanced lung cancer showed markedly higher serum CD3^+^, CD4^+^, and CD4^+^/CD8^+^ cell counts in the observation group than in the control group after treatment, accompanied by a better QOL (higher KPS scores) and fewer toxic and side effects (172). This study demonstrated that PG2 combined with gefitinib for advanced NSCLC can improve patients’ immunity, alleviate toxicity and side effects, and improve patients’ overall QOL. Zheng et al. showed that PG2 has a protective effect on the bone marrow, which can reduce the myelotoxicity of platinum-containing drugs combined with other drugs in patients with NSCLC and improve patient tolerance to chemotherapy (173).

Concurrent radiotherapy and chemotherapy are standard therapies for patients with advanced head and neck squamous cell carcinoma; however, the resulting complications can affect patient QOL. Moreover, its efficacy is low. Relapse and metastases are found in half of the head and neck squamous cell carcinoma cases, leading to a 5-year survival rate lesser than 40% (174). Hsieh et al. showed that although compared to the placebo group, patients administered PG2 did not show any difference in their tumor response rate, disease-specific survival rate, or overall survival rate, their QOL was significantly improved as indicated by the reduction in pain and improvement in appetite (175) (**Table 6**).

**Table 6:** Clinical trials of APS'anti-tumor effect.

Cancer type	Clinical Study Stage	Number of participants	Treatment plan	Dosage and treatment courses	Observation Results (↑upregulation, ↓downregulation)	REF
Lung cancer	Already on the market	53	Group 1: ICI treatment Group 2: ICI combined with PG2	500mg/d, 6 weeks (±2 weeks)	neutrophil-lymphocyte ratio↑	(170)
Non-small cell lung cancer	Already on the market	75	Group 1: CIK therapy Group 2: CIK therapy combined with PG2	250mg/d, 10d	CD4^+^ cells↑, CD3^+^ cells↑, Symptom of Qi deficiency↓	(171)
Non-small cell lung cancer	Already on the market	80	Group 1: Gefitinib Group 2: Gefitinib combined with PG2	250mg/d, 21d	CD4^+^ cells↑, CD3^+^ cells↑, CD4^+^/CD8^+^ cells↑ and adverse reaction rate↓	(172)
Non-small cell lung cancer	Already on the market	61	Group 1: CT Group 2: CT combined with PG2	250mg/d, 10d	Myelosuppression rate↓	(173)
Head and neck squamous cell carcinoma	Phase II clinical study	17	Group 1: CCRT Group 2: CCRT combined with PG2	500mg, tiw	adverse reaction rate↓, Pain relief and improved appetite	(175)

ICI, Immune checkpoint inhibitors; CIK, Cytokine-induced killer cells; CT, Chemotherapy; CCRT, Concurrent chemoradiotherapy.

Although these results are encouraging, owing to the limited number of patients and the influence of other potential factors, more comprehensive research is needed to ensure the optimal use of APS and its clinical safety profile in cancer therapy.

## 3 Discussion

The rising morbidity and mortality due to cancer in recent years have posed a serious threat to human health; however, its pathogenesis has not been thoroughly understood. The effectiveness of existing therapies is limited, and complete tumor treatment is difficult. Radiotherapy and chemotherapy not only cause pain and reduce the QOL but also increase the economic burden on patients. Currently, effective agents with low toxicity and few side effects need to be explored for treatment or adjuvant therapy.

**Figure 1:**
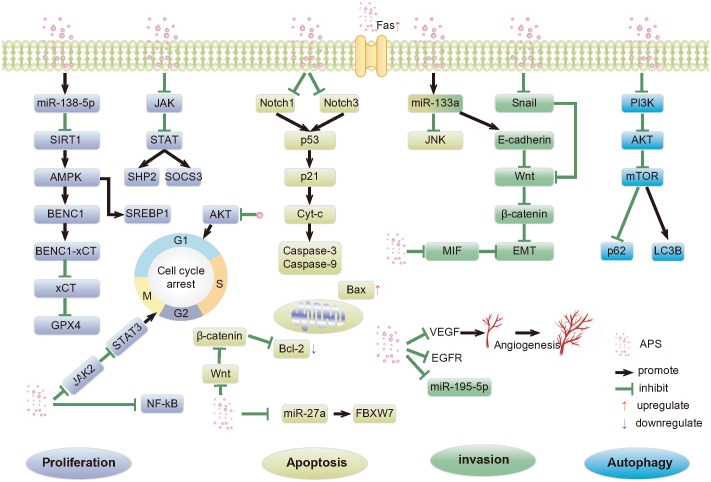
Anti-tumor pathways mediated by APS. APS, Astragalus polysaccharides; SIRT1, Silent mating type information regulation 2 homolog- 1; AMPK, Adenosine monophosphate activated protein kinase; SREBP1, Recombinant Sterol Regulatory Element Binding Transcription Factor 1; BECN1,Beclin-1; xCT, Light chain subunit SLC7A11; GPX4,Glutathione peroxidase 4; JAK, Janus kinase; STAT, Signal transducer of activators of transcription; SHP2, SH2 domain-containing protein tyrosine phosphatase 2; SOCS3, Suppressor of cy-tokine signaling proteins; AKT, Protein kinase B; NF-κB, Nuclear factor kappa-B; p53, p53 protein; p21, p21 protein; Cyt-c, Cytochrome c; Bcl-2, B-cell lymphoma-2; Bax, Bcl-2-associated X; FBXW7, F-box/WD repeat-containing protein 7; JNK, c-Jun N-terminal kinases; EMT, Epithelial-mesenchymal transition; MIF, Macrophage migration inhibitor factor; VEGF, Vascular endothelial growth factor; EGFR, Epidermal growth factor receptor; p63, p63 protein; PI3K, Phosphatidylinositol-3-kinase; mTOR, Mammalian rapamycin target protein; LC3B, Microtubule-associated protein 1 light chain 3 Beta.

As a natural product, APS has high medicinal value. It has multi-pathway, multi-target, and multi-level anti-cancer mechanisms. These include inducing apoptosis; regulating tumor cell autophagy; inhibiting tumor cell proliferation, invasion, and metastasis; tumor-related inflammatory microenvironment; and synergism with anticancer drugs (**Figure 1**). These mechanisms have broad research prospects, which warrant in-depth research.

Despite its promising benefits, the study of APS has some limitations. First, the potential mechanisms and causality of its active ingredients in complex cell signaling pathways are not comprehensively understood, and consistent models and evaluation criteria, particularly high-quality and large-sample clinical data, are lacking. Second, the literature survey showed that the APS dose used in related studies varied significantly. Therefore, the safe and effective dose of APS in follow-up studies should be determined, and a relatively safe regimen developed. Moreover, current research on APS is primarily limited to *in vitro* cell experiments and rodent models, and its optimal dose and safety need to be verified. Additionally, because APS is a hydrophilic macromolecular mixture, its bioavailability is low, and the combination of APS and nanocarriers can overcome this obstacle to achieve targeted drug delivery, effectively reduce drug dosage, and improve bioavailability. However, research on APS-modified nanocarriers to improve the specific targeting of tumor tissues is lacking.

In conclusion, APS has great potential in cancer therapy, particularly as nanoparticles obtained by APS processing. To establish the effectiveness of APS in clinical treatment and its broader anti-tumor mechanism and, thus, provide a more efficacious treatment for patients with cancer, further experimental exploration and research are essential.

## Author contributions

QY: Writing – original draft, Investigation. DM: Investigation, Writing – review & editing. QZ: Project administration, Supervision, Writing – review & editing. JW: Data curation, Supervision, Writing – review & editing.

## Glossary

**Table:**

APS	Astragalus polysaccharides
Bcl-2	B-cell lymphoma-2
Bax	Bcl-2-associated X
HCC	Hepatocellular cancer
c-myc	Myelocytomatosis viral oncogene homolog
Cyclin D1	G1/S-Specific cyclin-D1
BC	Breast cancer
JNK	c-Jun N-terminal kinases
TNF	Tumor necrosis factor
JAK	Janus kinase
STAT	Signal transducer of activators of transcription
AKT	Protein kinase B
AMPK	Adenosine monophosphate activated protein kinase
BECN1	Beclin-1
GPX4	Glutathione peroxidase 4
xCT	Light chain subunit SLC7A11
SIRT1	Silent mating type information regulation 2 homolog- 1
SREBP1	Recombinant Sterol Regulatory Element Binding Transcription Factor 1
SHP2	SH2 domain-containing protein tyrosine phosphatase 2
SOCS3	Suppressor of cy-tokine signaling proteins
NF-κB	Nuclear factor kappa-B
EMT	Epithelial-mesenchymal transition
MIF	Macrophage migration inhibitor factor
PG2	APS for injection
AXL	Recombinant AXL Receptor Tyrosine Kinase
NSCLC	Non-small cell lung cancer
VEGF	Vascular endothelial growth factor
EGFR	Epidermal growth factor receptor
HUVECs	Human umbilical vein endothelial cells
ROS	Reactive oxygen species
∆Ψm	Mitochondrial membrane potential
CDDP	Cisplatin
PD-L1	Programmed cell death protein-ligand 1
PI3K	Phosphatidylinositol-3-kinase
P-gp	P-glycoprotein
MMP	Matrix metalloproteinase
OGT	O-GlcNAc transferase
OGA	O-GlcNAcase
ERK	Extracellular Regulated Protein Kinases
IL	Interleukin
DC	Dendritic Cells
iNOS	Inducible nitric oxide synthase
NO	Nitric oxide
TNF	Tumor necrosis facto
MDSCs	Myeloid-derived suppressor cells
CD	Cluster of Differentiation
HIF-1	Hypoxia-inducible factor-1
MSN	Moesin
mTOR	Mammalian rapamycin target protein
P70S6K	P70 ribosomal protein S6 kinase
LC3B	Microtubule-associated protein 1 light chain 3 Beta
Beclin1	Bcl-2 interacting coiled-coil protein 1
ICI	Immune checkpoint inhibitors
Cyt-c	Cytochrome c
CIK	Cytokine-induced killer cells
